# A Comparative Study on Mechanochemically and Thermally Prepared Deep Eutectic Solvents

**DOI:** 10.1021/acsomega.5c01788

**Published:** 2025-05-28

**Authors:** Oluseyi Olawuyi, Md Rakibul Hasan, Tomasz Kruczyński, Abdul Hannan, Mohammad A. Halim

**Affiliations:** † Department of Chemistry and Biochemistry, 271594 Kennesaw State University, Kennesaw, Georgia 30144, United States; ‡ Division of Environmental and Green Chemistry, The Red-Green Research Center, BICCB, 16, Tejkunipara, Tejgaon, Dhaka 1215, Bangladesh

## Abstract

Recent developments in mechanochemistry have enabled the preparation of deep eutectic solvents (DESs) that can be a greener and viable alternative to the traditional thermal DESs. Since the mechanochemical method can be performed under ambient conditions, the mechanical energy generated during the grinding process can be sufficient to dissolve the solid HBA and HBD components and create a homogeneous mixture of DES. As thermally prepared DESs involve heating their components, often an extended duration of heating may alter their functionality through byproduct or covalent bond formation. In contrast, the mechanochemical preparation of DES requires no heating. This significantly reduces energy consumption and reduces the chance of the formation of any covalent bonds. In this study, we prepared three classes of DES using thermal and mechano-assisted rotary tumbler ball milling techniques without the application of any heat and analyzed them using multitechnique approaches, including physical, spectroscopic, statistical, and thermal analysis. Spectroscopic FT-IR and ^1^H NMR investigations were used to confirm the formation of DESs and to detect the presence of any impurities or byproducts in both methods. Principal component analysis (PCA) of the FTIR spectra data showed that both methods produced similar results for all of the DESs prepared. Thermal analysis of the DESs was performed by using differential scanning calorimetry (DSC) and thermogravimetric analysis (TGA). The physical properties of DES were measured for all three types of DESs. The results revealed that in all cases, even and almost for more challenging DESs, the mechanochemical-assisted rotary tumbler ball milling method showed unique advantages in comparison with traditional preparation processes, in terms of energy, performance, security, functionality, operating time, economics, and thus industrial-scale reality. This method can reveal the pathway to meet the industry’s new expectations for DES production and the future development of this green solvent-based process in different fields.

## Graphical abstract

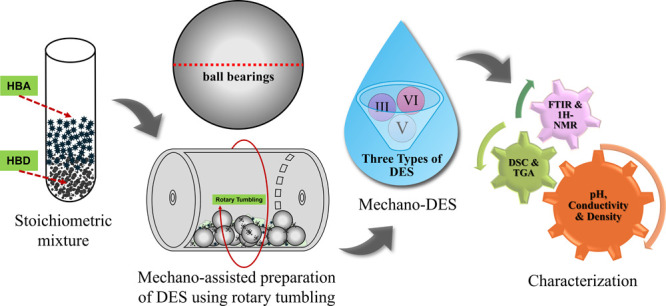

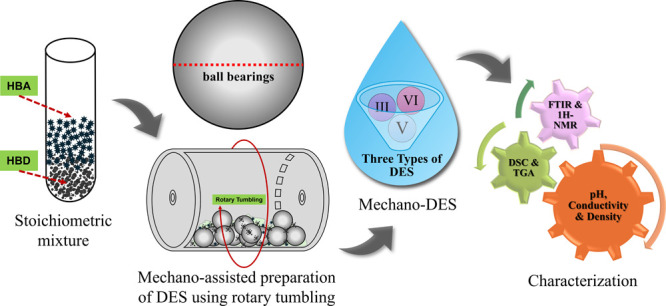

## 1 Introduction

Mechanochemistry is the application of mechanical energy to dissolve solid components and create a homogeneous mixture. Mechanochemistry in recent years has enabled the preparation of organic compounds, pharmaceutical compounds, organometallics, inorganic compounds, polymers, crystals, metal–organic frameworks, and nanomaterials.^1–3^ Since mechanochemical preparation can be performed under ambient conditions and does not require the application of heat, the components are mixed in a ball mill, mortar, and pestle, or other mechanical devices until they become finely ground and intimately mixed. The mechanical energy generated during the grinding process is sufficient to partially dissolve the solid components and create a homogeneous mixture.^4^ Mechanochemistry in recent years has emerged as a method for preparing deep eutectic solvents (DESs).^5,6^ Mechano-assisted preparation of DES requires no heating, which significantly reduces energy consumption and processing time, making it an economically suitable alternative to the thermal method.^7^

DES is currently viewed as a newer and more versatile alternative to ionic liquids (ILs), with a lot of benefits and widespread applications.^8^ DESs are classified as a novel type of solvents that are associated with hydrogen bond networks, followed by a depression in melting point at a fixed ratio when set against the individual constituents.^9^ The thermal method is the most common method for preparing DESs. Although this method is a fundamental and straightforward approach, it possesses some limitations and shortcomings. For instance, trying to synthesize DESs with a particular composition can be challenging using the thermal method, as the melting and freezing temperatures of the mixture can vary depending on the components used, their ratios and byproduct generated. Also, in the thermal method, the mixture of components is heated until they are completely melted, which can be energy-intensive and can elevate production costs. In certain cases, the reaction between the components may not be complete, leading to a mixture that is not a true eutectic mixture. This can negatively impact the stability and properties of the DES.^10,11^ Also, the thermal method may not be ideal for large-scale production or for preparing DESs with specific properties. Moreover, our previous investigation showed that thermal preparation resulted in the formation of multiple side products and failed to form noncovalent clusters in menthol-acetic acid DES, which was confirmed by mass spectrometry analysis.^12^ Mechanochemical preparation stands as a promising approach to overcome some, if not all, of these challenges that emanate from preparing DESs by using the thermal method. Mechanochemical synthesis is fundamentally distinct from thermal processes in that it employs mechanical forces like grinding and shearing to create localized amorphization, generate defects, and increase molecular mobility, enabling hydrogen bond formation without requiring significant heating.^13,14^ DESs formed through the mechanochemical method tend to show more homogeneous phase behavior and broader thermal transitions, indicating improved component miscibility, which can enhance their stability and usability in different applications.^13^ Mechanochemical preparation requires a simpler infrastructure, thus lowering capital and maintenance costs. Its scalability through ball milling or extrusion further supports its industrial relevance. The mechanochemical method provides a faster, cleaner, and more cost-effective route to DESs without compromising functional performance.^14^ Mechanochemical preparation of DES promotes hydrogen bonding under mild conditions, minimizing thermal degradation or covalent side reactions.^15^ Additionally, it ensures better reproducibility and scalability.^16^ However, mechanochemical reactions rely on high-intensity mechanical energy, often delivered through devices such as ball mills, which can result in significant localized energy usage. Although the overall process eliminates the need for sustained thermal input, the energy efficiency of milling operations may be lower than expected, particularly when scaled up due to frictional losses, inefficient energy transfer, and wear of milling components. Moreover, unlike thermal methods that scale linearly with volume in batch reactors, mechanochemical scaling is more complex.^16^ It often requires transitioning from batch ball milling to continuous extrusion or advanced milling systems that maintain consistent shear and pressure profiles across larger material volumes.^15^

Mechanochemical preparation of DES was studied earlier by others, but their studies did not incorporate the influence of synthesis parameters on DES properties.^17^ A recent relative study by Maruccho et al^6^ showed that mechanochemistry is a viable method of preparing deep eutectic solvents. Also in 2016, Abbott et al.^5^ demonstrated a novel method for the preparation of high-purity DES using twin screw extrusion. This method had been previously used for the extensive preparation of metal–organic frameworks (MOFs).^18^ In this study, we hypothesize that both mechanochemical and thermal preparation of DES affect physicochemical properties due to differing interaction mechanisms and energy inputs. Our study addresses this gap by examining how methods of preparation affect the ionic conductivity, and thermal stability of DESs. Previous literature discussed the sustainability of mechanochemical synthesis but did not compare it to traditional methods.^19^ We directly compare the mechanochemical method with a conventional thermal method for different classes of DESs. Our work adds to their findings by optimizing synthesis conditions for better physicochemical performance.

In our study, we highlight the diverse utility of the studied deep eutectic solvents (DES), which represent different categories based on their components. We used mechanochemical method with rotary tumbler ball milling to explore the preparation of three classes of DESs, including (i) Type III (ionic DES)—urea-choline chloride (2:1);^2,20^ (ii) Type V (Non-Ionic DES)—menthol-thymol (1:1);^21,22^ and (iii) Type VI (therapeutic DES)—Menthol-Ibuprofen (3:1);^23,24^ Which had been previously prepared by the thermal method. Urea-ChCl has found significant applications in various fields, including the extraction of bioactive compounds from natural sources, heavy metal removal, and even improving the mechanical properties of materials.^25–27^ The menthol-thymol-based DES has demonstrated potential in the extraction of nitrophenolic compounds, showcasing its application in environmental remediation and analytical chemistry.^28^ Furthermore, the menthol-ibuprofen DES, where one of the components is a drug or active pharmaceutical ingredient (API), has gained attention in pharmaceutical applications, particularly for enhancing the solubility and permeability of APIs for improved drug delivery.^29^ These examples underscore the tangible, practical significance and application value of exploring and understanding the properties of the different DES system types. The three classes of DESs were chosen for their frequent report, broad use, and industrial application. Our aim is to capture a wide spectrum of physicochemical behaviors while maintaining experimental feasibility. The process was optimized by employing ball milling of various diameters (10–16 mm).

## 2 Materials and Methods

### 2.1 Materials

DL-Menthol (Men; purity >98%; crystal), urea (purity >99.3%; crystal), thymol (Thy; purity >99%, crystalline powder), and choline chloride (ChCl; purity >99%; adhering crystals) were purchased from Thermo Fisher Scientific (MA, USA) and ibuprofen (Ibu; purity >97.0–103.0%; needle shape crystal) was purchased from Spectrum Chemical Mfg. Corp. (NJ, USA). All the chemical substances used for this study were of analytical grade, and their structural formulas are represented in Figure 1. The chemicals were kept in a vacuum chamber to avoid gaining any water in the samples and used without further purification.

**Figure 1:**
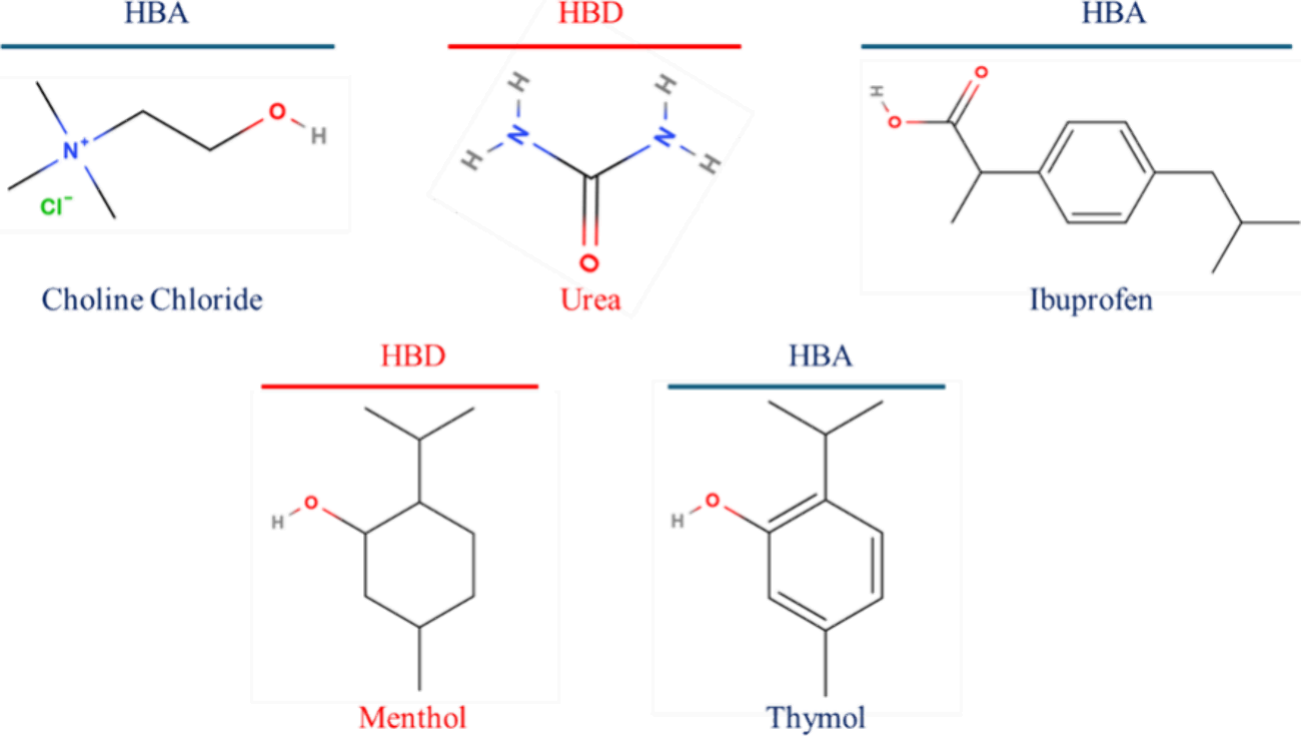
Chemical constituents employed in this study are shown with their respective molecular structures.

### 2.2 Preparation of the DESs by the Mechanochemical Method

Three classes of DESs including (i) Type III—urea-choline chloride (Figure S2—2:1 ratio); (ii) Type V—menthol-thymol (Figure S3—1:1 ratio); and (iii) Type VI—menthol-ibuprofen (Figure S4—3:1 ratio), were prepared using the mechanochemical method due to their prevalence and broad relevance across the current literature. These types are among the most frequently reported and industrially explored DES classes, encompassing hydrogen bond donors (HBDs) and acceptors (HBAs) with diverse chemical characteristics. After the solids were mixed in a small beaker, the mixture was transferred into a small cylindrical polypropylene plastic container. The size of the plastic container was 37 mm in diameter and 42 mm in height, with an airtight cap. The container size was chosen considering the size of the tumbler barrel and the synthesis of a small amount of DES in the laboratory within a shorter period. Various numbers (5, 10, 20, and 30) of ball bearings (15 mm diameter) were also tested (Figure 2). Although a smaller number of bearing balls can synthesize DES, they will generally take a longer time. Furthermore, a greater number of ball bearings makes it crowded, leaving less space for the sample, considering the size of the container. Similarly, a significant amount of time was required when smaller ball bearings (less than 10 mm in diameter) were employed. The menthol: ibuprofen DESs, synthesized using different numbers of ball bearings (15 mm), were analyzed by FTIR, and the presence and position of all major functional groups confirm that there is no significant impact on the property of DES due to variations in the ball bearing number (Figure S6). Low tumbler speed (100 rpm) also takes a longer time to prepare the DES. After several trials with different numbers of ball bearings and variations in rotation speed, finally the process was optimized by selecting 15 ball bearings of 15 mm diameter along with a tumbler speed of 3 (300 rpm). The container with its constituents was placed inside the rotary tumbler and rotated at tumbler speed 3 for 1–3 h until a homogeneous, clear liquid was obtained. In the mechanochemical preparation of Urea-ChCl DES, after mixing the solids in the tumbler, the mixture was transferred into a small mortar and ground for 20 min in the fume hood to obtain a homogeneous, clear liquid. The mortar and its contents were covered during the grinding to prevent moisture from interfering with the process. After the formulation, the DES was transferred to an airtight container and kept in the desiccator for further use. Each DES sample was synthesized more than 3 times by both mechanochemical and thermal methods to confirm the reproducibility of the process. The amount of the components required to synthesize each DES is mentioned in Table S1.

**Figure 2:**
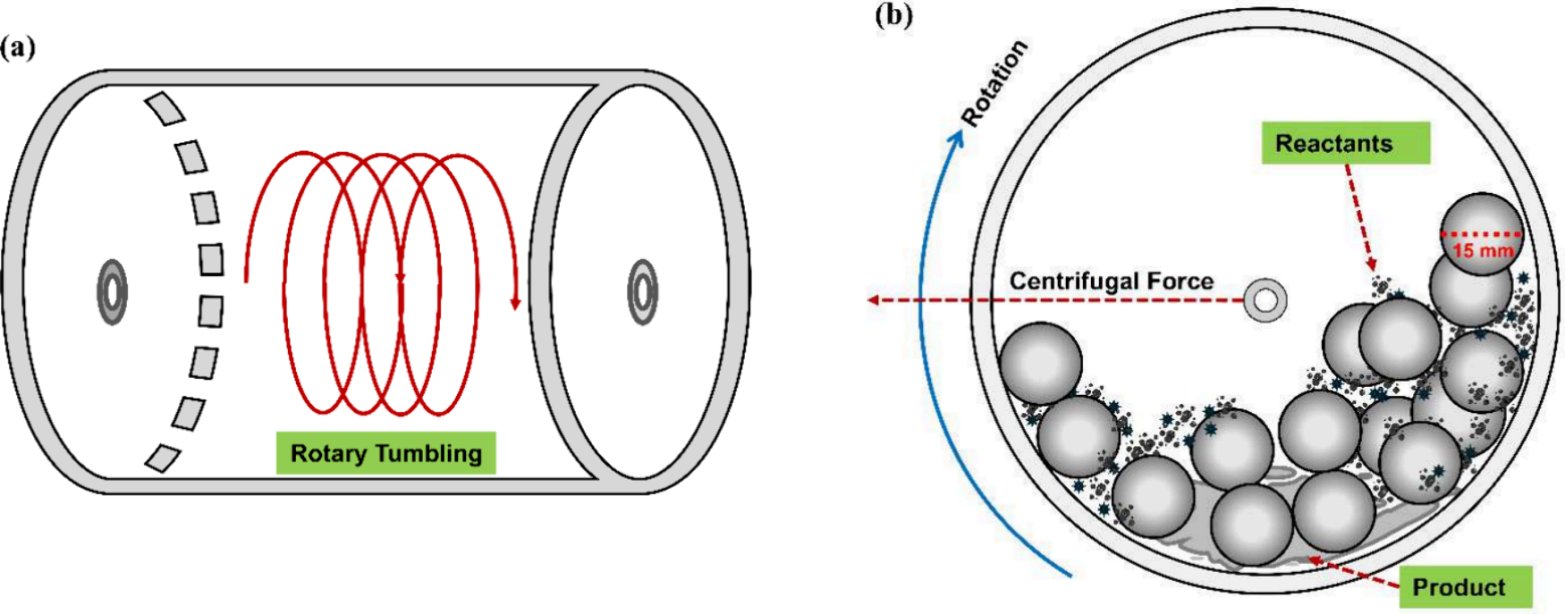
(a) Schematic representation of laboratory-scale rotary tumbler ball milling. Reactants are inserted in a small cylindrical polypropylene plastic container; (b) scheme of the rotation of the container, and inner section of a container. The reaction occurs in a closed vessel, containing the reactants and 5–20 pieces of ball bearings (10–16 mm in diameter each).

### 2.3 Attenuated Total Reflectance-Fourier Transform Infrared Spectrometer (ATR-FTIR)

In this study, an ATR-FTIR Spectrometer was used to characterize all three types of DES samples. A PerkinElmer Frontier FTIR instrument equipped with an attenuated total reflection module was used to obtain spectral data for samples analyzed in this study. Spectra were recorded at ambient temperature in the range of 500–4000 cm^–1^. Samples were recorded using 100 scans with a resolution of 16 cm^–1^, and the spectra were then processed using the PerkinElmer Spectra IR software suite and Origin software.

### 2.4 Principal Component Analysis (PCA)

Principal component analysis (PCA) is a statistical technique used as a dimensionality-reduction technique by diminishing noise and emphasizing the variabilities present in multivariate spectra of various chemical constituents.^30^ PCA diminishes the dimensionality of the information to explain the variance-covariance structure of a set of variables through linear combinations. PCA decomposes a data set with multiple variables in an *X* matrix into two new matrices using the following equation:

$$X=T_{k}{P_{k}}^{T}+E$$

In this equation, *T_k_* is the matrix of scores reflecting the correlations between samples, *P_k_* is the matrix of loadings which encapsulates information regarding the interrelations among variables, *k* is the number of components included within the model, and *E* is the network of residuals containing unexplained information.^12,31^ PCA calculations of the FTIR data sets were performed for all three DESs with Origin Pro (2022) equipped with the Principal Component Analysis for Spectroscopy v.1.30 Application.^32,33^

### 2.5 Experimental ^1^H Nuclear Magnetic Resonance (NMR)

The NMR spectra were collected at 298 K using a Bruker Avance III 600 spectrometer (600.2 MHz (^1^H) ). Chemical shifts are referred to the residual solvent peak (dimethyl sulfoxide-d_6_: (^1^H) 2.500 ppm according to the IUPAC recommendations.^34,35^ The data were processed with MestReNova software.

### 2.6 Differential Scanning Calorimetry (DSC)

A DSC 60 apparatus from SHIMADZU Corporation, Japan, operated in the heat flux system, was used for the experiments.^36,37^ Under an argon atmosphere and with the help of a liquid-nitrogen cooling system, standard calibration procedures were conducted with indium at a flow rate of 35 mL min^–1^. The samples were placed in aluminum pans and sealed in an airtight manner at a dosage of approximately 10 mg per sample. The samples were equilibrated at 35 °C for 5 min before cooling from 35 °C up to −130 °C, followed by an isothermal period of 5 min at −80 °C, and then heated from −80 to +100 °C at the rate of 5 °C min^–1^, and thereafter cooled to room temperature.

### 2.7 Thermogravimetric Analysis (TGA)

The thermogravimetric analyses of the DES samples were carried out using TGA (TGA 8000 coupled with TL 8000 system) from PerkinElmer Instruments, USA.^38,39^ Approximately 10 mg of samples of urea-ChCl, Men-Thy, and Men-Ibu DES were taken in the TGA aluminum pan for the analysis in each case. Both thermally and mechanochemically prepared samples were analyzed. The samples were initially conditioned at 25 °C for 5 min before being subjected to a constant heating rate of 5 °C/min as the oven temperature was raised from 25 to 300 °C under a nitrogen atmosphere with a flow rate of 70 mL/min, to maintain an inert atmosphere and prevent the sample from coming in contact with the air. In addition, nitrogen gas sweeps out pyrolysis gases like CO_2_, H_2_, CO, CH_4_, and water vapor because these gases interact with samples during the vapor phase. The experiments were performed in duplicate for each sample.

### 2.8 P^H^, Density, and Conductivity Measurements

The required amount of DES sample was weighed and dissolved in a suitable solvent to prepare a 0.5 mol/L solution.^40^ Menthol-thymol and menthol-ibuprofen DESs were dissolved in 100% ethanol, as the components are insoluble in water, whereas choline chloride-Urea DES was dissolved in deionized water. The freshly prepared solution was kept at room temperature for 30 min, and the pH of the solution was measured using a calibrated pH meter from Fisher Scientific (Model no: AB150). The conductivity of the 0.5 M DES solution was measured by using a YSI 3100 conductivity instrument.

## 3 Result and Discussion

### 3.1 ATR-FTIR Spectroscopy

ATR-FTIR spectroscopy was used to characterize the prepared DESs and examine the chemical changes in both thermal and mechanochemical methods, concerning their individual constituents. Figure 3a–c shows the ATR–FTIR spectra of all three classes of DES. In Figure 3a, the doublet peak seen around 3334–3423 cm^–1^ in urea is attributed to N–H stretching, while the peak around 3222 cm^–1^ in choline chloride is attributed to the O–H stretching. A similar but broadened doublet peak to what was seen in the urea FTIR spectrum was also seen in both thermally and mechanochemically prepared DESs around 3321–3197 and 3322–3194 cm^–1^, respectively. The broadening and subsequent shifting of the doublet peaks to the lower IR region in both DESs suggest the influence of the intermolecular hydrogen bonding between the N–H in Urea and the O–H group in choline chloride, as the intermolecular hydrogen-bonded O–H stretching is usually observed in this region. There is also a possibility of intermolecular hydrogen bonding between N–H in urea and the Cl^–^ ion in choline chloride. The N–H bending vibration seen around 1592 cm^–1^ in urea is also broadened and shifted to 1597 and 1598 cm^–1^ in the thermally and mechanochemically prepared DESs. The IR spectra of both thermal and mechanochemically prepared DESs are identical. This is an indication that mechanochemical preparation may also be used to prepare urea-choline chloride DES with success.

**Figure 3:**
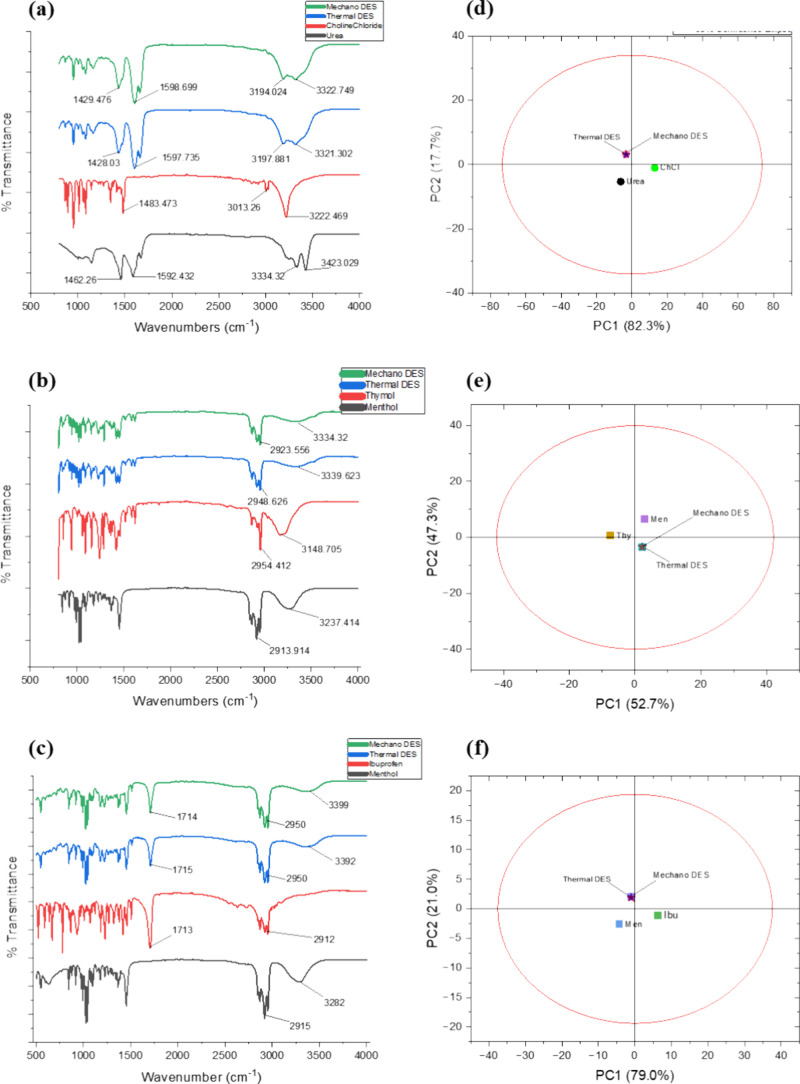
FTIR spectra (a–c) and PCA score plots (d–f) from the FTIR spectral data for urea, choline chloride, thermal, and mechano-DES (top); menthol, thymol, thermal, and mechano-DES (middle); and menthol, ibuprofen, thermal, and mechano-DES (bottom).

For Men-Thy DES, in Figure 3b, the peak around 2913 cm^–1^ is caused by C–H stretching, whereas the peak at around 3237 cm^–1^ is caused by O–H stretching of the O-H functional group. Thymol exhibits O–H stretching at 3148 cm^–1^ as well, whereas the several peaks at 2954 cm^–1^ can be a result of C–H stretching vibrations.^41^ The development of DESs between the two compounds is most likely caused by hydrogen bonding, as seen from a comparison of the chemical structures of menthol and thymol. Other forces that may also play roles in the stability of the DES system include van der Waals and Π–Π interaction, due to the aliphatic and aromatic rings in menthol and thymol, respectively.^42,43^ The O-H peak positions in both menthol and thymol overlapped, broadened, and shifted in both thermal and mechanochemically prepared Men-Thy DES. In both cases, the O-H peak is seen around 3339 and 3334 cm^–1^, respectively. Relatively less-intense C–H vibrations are also seen around 2948 and 2923 cm^–1^ in thermally and mechanochemically prepared DESs, respectively. These results stand as evidence that both thermal and mechanochemical approaches may be used to prepare Men-Thy DES, as the functional group region and the fingerprints region of the FTIR spectra of the DESs prepared by both methods showed close similarity.

For Menthol-Ibuprofen DES, in Figure 3c, the noticeable peak seen around 3282 cm^–1^ in Menthol is due to the O–H stretching of the O-H functional group, while the peak around 1713 cm^–1^ in Ibuprofen represents the C=O stretching of the carbonyl group. Since the formation of a DES is usually due to the intermolecular hydrogen bonding and other noncovalent interactions between the HBD (menthol in this case) and the HBA (ibuprofen in this case), these two functional groups are crucial to the formation of the expected Men-Ibu DES as they are responsible for the formation of hydrogen bonding, and dipole–dipole interactions in the DES system.^43,44^ Based on the FTIR data, the first evidence that a DES was formed between the two solids was the appearance of both the O-H peak seen in menthol, and the C=O peak seen initially in ibuprofen, in the Men-Ibu DES. As expected, there was a shift in the positions of these two key functional groups in the resulting DES. The O–H stretching was broadened and shifted from the initial position of 3282 cm^–1^ in Menthol to 3392 cm^–1^ in thermal DES, and 3399 cm^–1^ in the DES made by a mechanochemical method due to the formation of hydrogen bonding in the DES. The slight shift in the position of the C=O group from 1713 to 1715 cm^–1^ in thermal DES, and 1714 cm^–1^ in the mechanochemically prepared DES, with noticeable reductions in the intensity of the peak in both cases. This may be attributed to noncovalent interactions between menthol and ibuprofen that resulted in the formation of Men-Ibu DES. The C–H stretching vibrations that are present in both menthol and ibuprofen also appeared at 2950 cm^–1^ in both DESs. These results revealed that the DES prepared by both thermal and mechanochemical methods had identical IR spectra. This suggests, to a reasonable extent, that mechanochemical preparation may be another viable approach to the preparation of ibuprofen-menthol DES, although further analysis using other characterization approaches may be required to substantiate this claim.

Figure 3d–f represents the PCA score plot of the FTIR data sets of Urea-ChCl, Men-Thy, and Men-Ibu DESs, respectively, prepared by thermal and mechanochemical methods, with respect to their constituents were performed (Figure 3).^8^ The variations in the spectra data were described by two principal components (PC1 and PC2) for all three types of DESs. Since the first principal component explains the higher variance and the second principal component (PC2) explains decreased amounts of variance, the similarities and variations between the four spectra data for each DES may be better defined along the PC1 axis (*x*-axis).^45,46^ Interestingly, for urea-ChCl, the total variation described by both PCs was 100% (82.3 and 17.3% for PC1 and PC2, respectively).

### 3.2 Principal Component Analysis

From the scores plot, the scores of the urea-ChCl DES made by both thermal and mechanochemical methods overlapped completely. This means that both DESs have identical IR spectral data sets, which is evidence that the two methods of DES preparation produced similar results. Also, the scores of both DES are positioned between the scores of urea and choline chloride, although they are closer to the scores of urea. This means that both urea and choline chloride contributed to the formation of the DESs, but the concentration of urea, which invariably represents its contribution to the formation of the DES, is more than that of choline chloride in the DES. This result is consistent with the stoichiometry of the components used in the preparation of the DESs [Urea-ChCl (2:1)].

In the case of Men-Thy DES, the total variation described by both PCs was 100% (52.7 and 47.3% for PC1 and PC2, respectively). Since the margin between the values of PC1 and PC2 is not far apart, the similarities and variations between the four spectra data may be defined by both PC1 and PC2.^45^ From the scores plot, the scores of the Men-Thy DES prepared by both the thermal and mechanochemical methods overlapped, which suggests that both DESs have identical IR spectra data sets, which evidence that the two methods of DES preparation produced similar results. Also, the PC scores of both DES are positioned between the scores of thymol and menthol, although closer to the score of menthol. This indicates that both thymol and menthol contributed to the formation of the DESs, but the menthol component is more represented in the DES. Since the stoichiometry of the components used in the preparation of the DESs is 1:1 [Thy: Men (1:1)], the larger molar mass of menthol may be responsible for its dominance.

For Men-Ibu DES, the total variation described by both PCs was also 100% (79.0 and 21.0% for PC1 and PC2, respectively). From the score plot, the scores of the Men-Ibu DES made by both thermal and mechanochemical methods overlapped, which suggests that both DESs have identical IR spectra data sets, which is evidence that the two methods of DES preparation produced similar results. Also, the PC scores of both DES are positioned between the scores of ibuprofen and menthol, although closer to the score of Menthol. This means that both ibuprofen and menthol contributed to the formation of the DESs, but the Menthol component is more represented in the DES. This result is consistent with the stoichiometry of the components used in the preparation of the DESs [Ibu: Men (1:3)].

### 3.3 ^1^H Nuclear Magnetic Resonance (NMR)

Due to the significantly different reaction temperature, different byproducts should be expected in thermally induced and mechanochemical reactions. The composition of DESs has been investigated with ^1^H NMR spectroscopy. Figures 4a,b and S1 represent the ^1^H NMR experiments for Urea-ChCl, Men-Ibu, and Men-Thy DESs, all of which confirmed satisfactory purity of the individual compounds, i.e., urea, choline chloride, menthol, thymol, and ibuprofen. Moreover, these data are a comparator in further analysis. Significant relative shift of the corresponding ^1^H NMR resonances is observed compared with the spectra of individual compounds. This feature can be attributed to the change of the H-bond network in the solution.^47^ The resonance signals of the expected compounds in DESs have been identified. Within the limits of the method, regardless of the preparation process, all samples present similar levels of purity. The exception is the reaction between chloride and urea in a 1:2 molar ratio. In contrast to mechanochemical synthesis, the ^1^H NMR spectrum of the sample from the thermal reaction presents additional resonance signals: a partially overlapping signal at 5.58 ppm and a singlet at 3.48 ppm. These signals are not present in the case of mechanochemical synthesis and indicate the higher selectivity of the nonthermal synthesis. Mechanochemical synthesis leads to products of higher purity.

**Figure 4:**
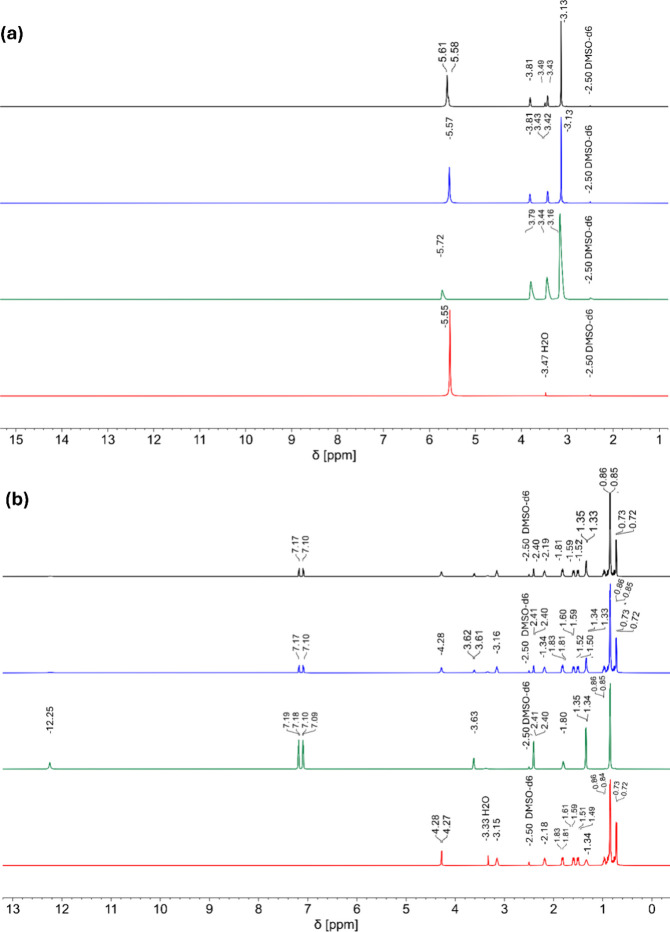
(a) ^1^H NMR spectrum shown in red denotes urea, green denotes choline chloride, blue denotes mechano DES (1:2), and black denotes thermal DES (1:2), DMSO-d6, 298.1 K, (^1^H) 600.2 MHz); (b) ^1^H NMR spectrum shown in red denotes menthol, green denotes ibuprofen, blue denotes mechano DES (1:1), and black denotes thermal DES (1:1), DMSO-d6, 298.1 K, (^1^H) 600.2 MHz).

### 3.4 Differential Scanning Calorimetry (DSC)

The lowering of the melting point of the prepared DES depends upon its individual HBD and HBA constituents.^11^ DSC was performed to further study all three types of DES prepared by both thermal and mechanochemical methods.^48^ Figures 5a,b and S2 represent the DSC heat thermogram for the prepared DESs. Analyzing DSC data for the temperature range studied revealed phase transitions within the samples.^49^ No endothermic transition was observed in the thermogram of both thermal and mechanochemical natures for the prepared DESs. For urea-ChCl DES (Figure 5), in the thermal method, the onset of exothermic transitions at 11.01 °C, which peaks at 3.17 °C, suggested that the crystallization of the DES’s components started in this temperature range, and its use as a liquid below these temperatures may not be effective. Subsequent peaks after these temperatures were less conspicuous. For the mechanochemical Urea-ChCl DES, the onset of the exothermic transition was observed at 21.30 °C. Other exothermic peaks were seen after this temperature, notably around 21.15 °C and below. These peaks may be attributed to the freezing of the mechanochemical DES. Although the urea-ChCl DES exhibits irregular exothermic transitions in both cases, there is a close similarity in their patterns. The difference in the onset of crystallization in both cases may be attributed to the differences in their uniformity. While mechanochemistry may be considered a promising route for urea-ChCl DES preparation, further optimization of the process may be required to create a more uniform urea-ChCl DES system. For Men-Thy DES (Figure S2), endothermic peaks which signify melting, were seen at 58.82 °C for thymol, and 35.74 °C for menthol. These peaks agree with the theoretical melting points of both solids. In the thermal Men-Thy DES, exothermic transition peaks were noticed at 4.70 and −43.15 °C. The peak at 4.70 °C may be attributed to the crystallization of some of the particles of either of the components, while the peak at −43.15 °C may suggest the freezing point of the DES. Mechanochemically prepared Men-Thy DES has an exothermic transition peak at – 42.19 °C. This peak signifies the freezing point of the DES. The DES prepared by both thermal and mechanochemical methods has almost the same freezing point, which supports the fact that mechanochemistry may be a promising method of preparing Men-Thy DES, just like conventional thermal methods. The single exothermic peak found in the mechanochemical DES is also an indication that the method produced a more uniform DES system than the thermal method in this case. For Men-Ibu DES (Figure 5), endothermic peaks, which signify melting, were seen at 77.72 °C for ibuprofen and 35.74 °C for menthol, respectively. These peaks agree with the theoretical melting points of both solids. As expected of a stable liquid, no endothermic transition was observed in the thermogram of Men-Ibu DES. In the thermal Men-Ibu DES, the onset of exothermic transition at 25.63 °C, which peaks at 31.87 °C, may be attributed to the crystallization of some of the Menthol particles, as there are excess menthol particles in the DES system. A similar trend is observed in the mechanochemical Men-Ibu DES, but the exothermic transition peaks at 30.44 °C. Analysis of the mixtures containing menthol was limited by the evaporation of menthol at a higher temperature; therefore, the thermogram presented corresponds to the first heating run. The DSC curves for all of the mechanochemically prepared DESs generally display broader and less distinct thermal events compared to the thermal DESs. This suggests that the DESs synthesized by mechanochemical methods may possess a less organized molecular network, potentially due to incomplete or less extensive hydrogen bonding during solid-state mixing. The absence of a strong, well-defined crystalline structure or organized molecular arrangement could weaken thermal stability slightly.^50,51^ Therefore, the lower decomposition temperatures observed in mechanochemical DESs can be attributed to a less tightly bound hydrogen-bonding network and higher structural disorder compared with the thermally prepared DESs.

### 3.5 Thermogravimetric Analysis (TGA)

The thermal stability of the DESs is determined by the decomposition temperature (*T*_dcp_) evaluated from TGA analysis. Decomposition temperature is the temperature at which the DES sample loses 10% of its original weight during thermogravimetric analysis.^50^ Figure 6a–f represents the dynamic TGA curves of all three types of DESs, including their constituents, prepared in thermal and mechanochemical methods. The decomposition temperatures for all DESs and their molar ratios are also represented in Table 1. It is evident from Figure 6 that the TGA curve for mechanochemical DES followed the same trend as that of thermal DES, with a slightly lower *T*_dcp_ value (Table 1), particularly in the case of urea-ChCl (167.71 °C) and Men-Ibu (106.86 °C) DESs. Since *T*_dcp_ represents the maximum temperature at which the DES can remain in the liquid state and retain its properties without decomposition, it may be concluded that the mechanochemical DES may exhibit slightly less thermal stability than the DES prepared by the thermal method.^6^ In both methods of urea-ChCl DES preparation, the TGA curve of the DES is found between the TGA curves for urea and ChCl. A similar trend has been previously reported in the literature.^50^ The slightly lower decomposition temperature observed in mechanochemically synthesized DESs, compared with their thermally prepared counterparts, may be attributed to differences in the microstructure and hydrogen bonding networks arising from the preparation method. Mechanochemical synthesis, being solvent-free and rapid, often results in incomplete or less-ordered hydrogen bond networks due to limited molecular mobility during grinding. In contrast, thermal preparation allows extended molecular interactions under heat, leading to more stabilized supramolecular assemblies and stronger hydrogen bond networks, which can enhance the thermal resistance. Additionally, previous reports have shown that DESs formed under thermal conditions may exhibit a partial covalent character or deeper eutectic behavior, contributing to enhanced thermal stability. The lack of thermal equilibration in the mechanochemical route likely results in metastable or kinetically trapped states with lower energy barriers for decomposition.

**Figure 5:**
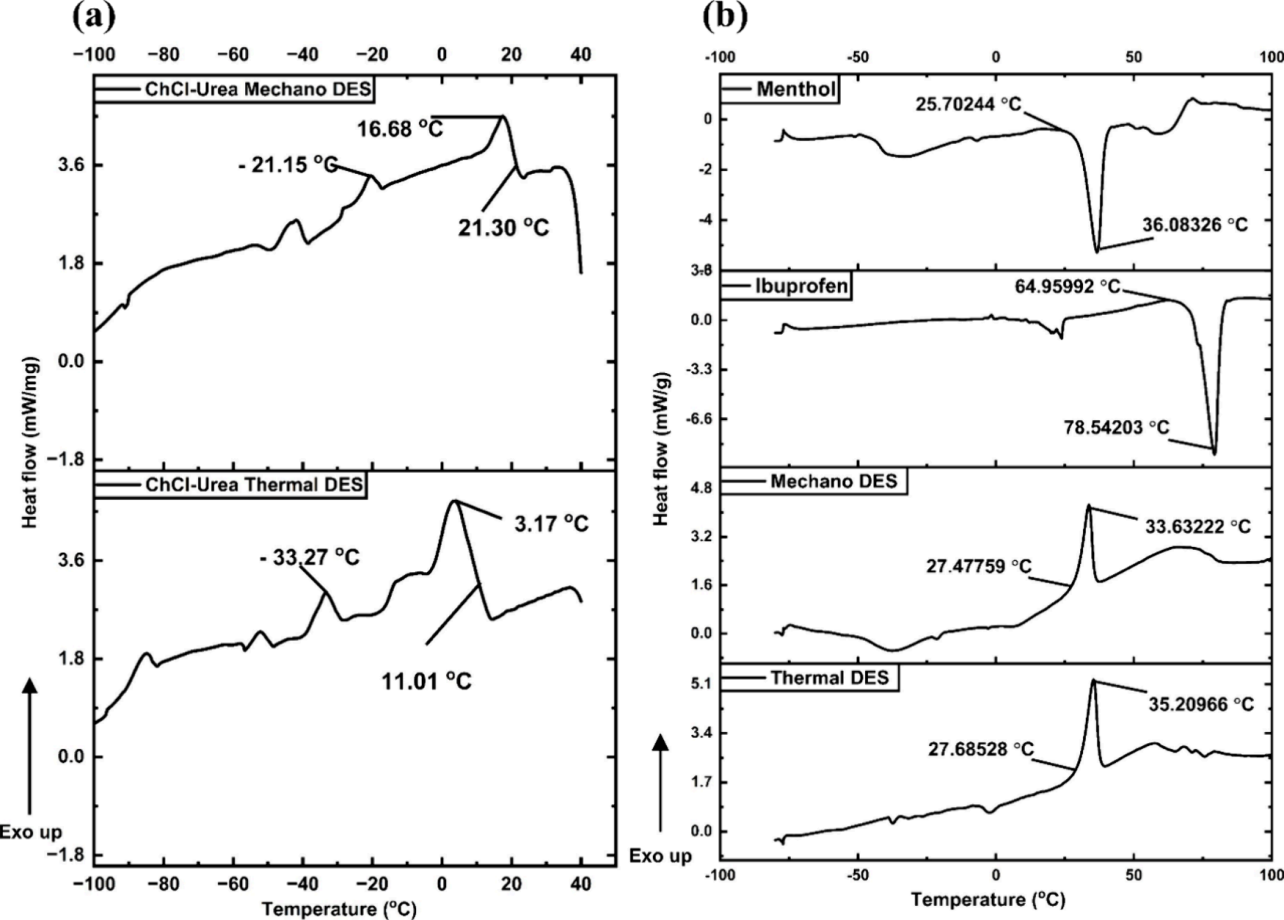
DSC Thermograms for (a) urea-ChCl mechano-DES and thermal-DES (left) and (b) menthol, ibuprofen, mechano-DES, and thermal-DES of Men-Ibu [reproduced from ref 37 with permission from the Royal Society of Chemistry] (right).

**Table 1:** Decomposition Temperature (*T*_dcp_) for All Three Types of DESs along with Their Individual Constituents and Molar Ratio in Both Thermal and Mechanochemical Methods

			*T*_dcp_/°C		
preparation method	DESs (HBD:HBA)	molar ratio (HBD:HBA)	DES	HBD	HBA
thermal	urea:ChCl	2:1	173.85	180.49	293.00
	Men:Thy	1:1	116.79	93.73	110.80
	Men:Ibu	3:1	108.15	93.73	175.40
mechano-chemical	urea:ChCl	2:1	167.71	180.49	293.00
	Men:Thy	1:1	116.50	93.73	110.80
	Men:Ibu	3:1	106.86	93.73	175.40

For Men-Thy DES, the *T*_dcp_ for mechano DES was almost identical to that of the Thermal DES, as it was seen around 116.50 °C. Since *T*_dcp_ represents the maximum temperature at which the deep eutectic solvent can remain in the liquid state and retain its properties without decomposition, it signifies the temperature range under which their application as a solvent is feasible.^6^ Here, the *T*_dcp_ value of the Men-Thy DES is greater than the *T*_dcp_ value of its individual components. This is an indication that the DES is more thermally stable than both of its components. The similarity of the dynamic TGA curves for both thermal and Mechano DES, and their nearly identical *T*_dcp_ values, suggests that both methods produced DES of similar composition. This result presents mechanochemistry as a viable alternative synthetic method for Men-Thy DES preparation.

For Men-Ibu DES, the *T*_dcp_ for Mechano DES was found around 106.86 °C, which is greater than the *T*_dcp_ value of Menthol but less than the *T*_dcp_ value of Ibuprofen. This indicates that Men-Ibu DES is more thermally stable than menthol (which has a lower melting point of the two components of the DES), but less thermally stable than Ibuprofen. This is a common trend in the literature in which the *T*_dcp_ of the DES is seen between the *T*_dcp_ values of its constituent compounds.^50^ The dynamic TGA curves for both thermal and mechano DES are similar, but their *T*_dcp_ values revealed that the Men-Ibu DES made by the thermal method is more thermally stable than the mechanochemically prepared DES (Figure 6).

**Figure 6:**
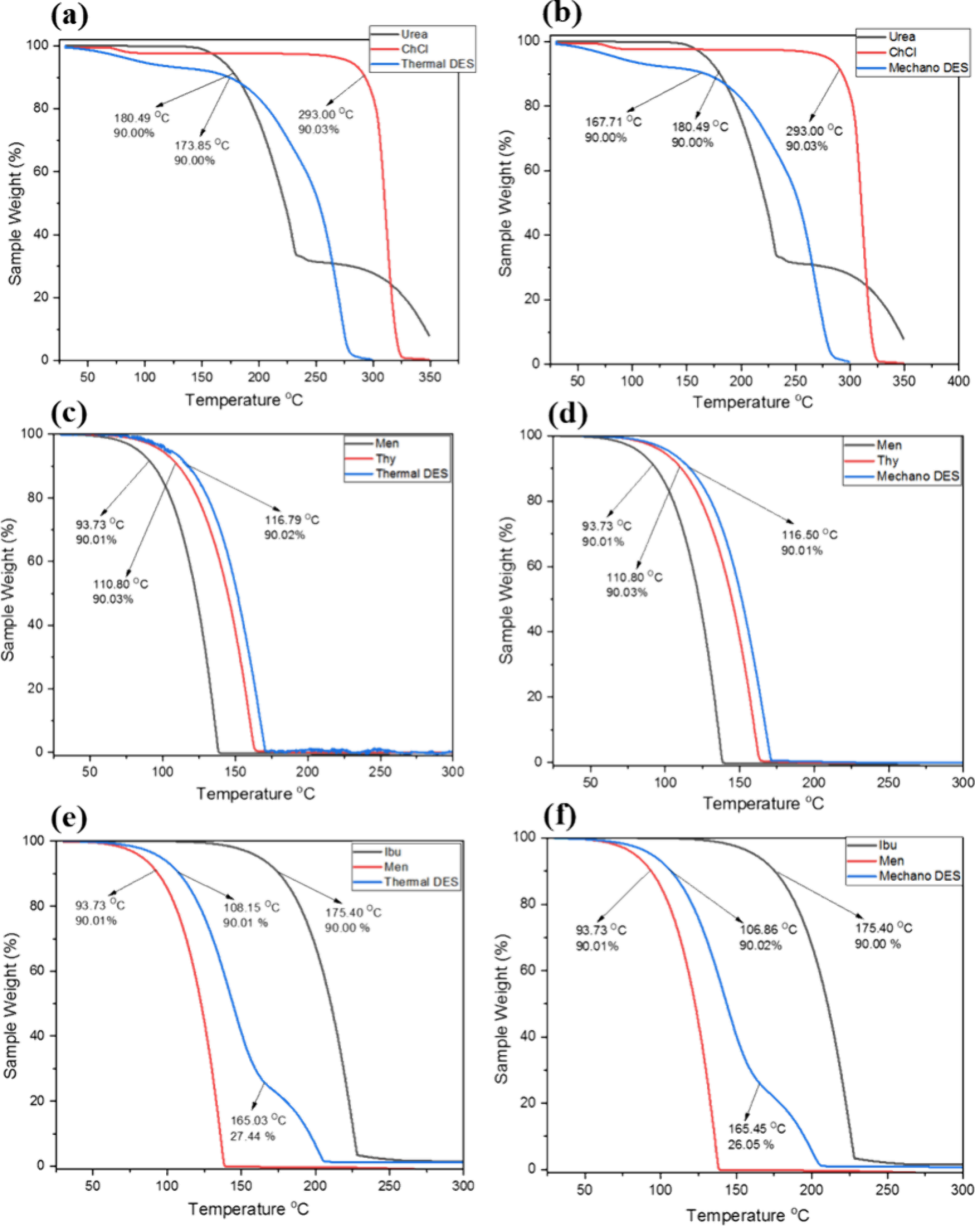
Dynamic TGA thermograms for (a, b) urea, choline chloride, thermal, and mechano-DES (top), (c, d) menthol, thymol, thermal, and mechano-DES (middle), and (e, f) menthol, ibuprofen, thermal [reproduced from ref 37 with permission from the Royal Society of Chemistry], and mechano-DES (bottom).

### 3.6 Comparison of pH, Density, and Conductivity of DESs

The pH and Density of DES are key physical properties having potential impact on applications in biochemical interaction and biocatalysis. Studies by Hayaan et al. and Kareem et al. showed that the type of HBD and HBA controls the acidity and the basicity of DES. The pH of DES decreases as the HBD content is reduced.^52–54^ Density has a crucial impact on solvation ability, mass transfer rate, extraction, absorption, and separation capacity of DES.^55^ Conductivity of DES is also considered an important characteristic. The electrical conductivity and thermal conductivity of DES can be critical for specific applications.^56,57^ Understanding and controlling the density of DESs are essential for optimizing their use in various industrial applications. Temperature, molar ratio of component, and water content can significantly affect the pH, density, and conductivity of DES.^55,58,59^ In this study, we compared the physical properties of three DESs prepared by the thermal method and the mechanochemical method. For Urea-ChCl DES, the pH was observed to be 8.73 and 4.43 for thermal and mechano DES, respectively. The density of thermal DES is found to be 1.7760 g/cm^3^, which is similar to that of mechano DES (1.7430 g/cm^3^). No significant difference observed in conductivity results between thermal DES (27,000 μs/cm) and mechano DES (26,500 μs/cm) (Table 2). For Men-Thy DES, almost similar pH, density, and conductivity were observed in thermal and mechano methods. The pH of thermally prepared DEs was 6.63, and the pH of mechano-DES was 6.38. There was no noticeable difference observed in the density of thermal DES (1.8037 g/cm^3^) and mechano DES (1.8166 g/cm^3^). Almost similar conductivities, 26,890 and 27,190 μs/cm, were observed for thermal and mechano DES, respectively. For Men-Ibu DES, the pH was almost identical, 4.38 and 4.37 in thermal and mechano DES, respectively. The density of thermal DES was found to be 1.0564 g/cm^3^, whereas the density of mechano DES was 1.0872 g/cm^3^. The thermal DES exhibited conductivity at 10.12 μs/cm, which was close to that of mechano DES at 11.48 μs/cm. The results obtained indicate that there is no significant difference between the pH, density, and conductivity of the mentioned DESs prepared in different methods. The physical properties of DES are well preserved in the mechanochemical method compared to the thermal method. Therefore, it can be concluded that the mechanochemical method could be a greener alternative to the traditional thermal method for the synthesis of DESs.

**Table 2:** Comparison of pH, Density and Conductivity of the Prepared DESs

	pH		density (g/cm^3^)		conductivity (μs/cm)	
DESs	thermal	mechanochemical	thermal	mechanochemical	thermal	mechanochemical
urea-choline chloride (2:1)	8.73	8.43	1.7760	1.7430	27,000	26,500
menthol-thymol (1:1)	6.63	6.38	1.8037	1.8166	26,890	27,190
menthol-ibuprofen (3:1)	4.38	4.37	1.0564	1.0872	10.12	11.48

## 4 Conclusions

In this work, a series of natural, environmentally friendly, and low-cost DESs have been prepared using both mechanochemical and thermal preparation methods. Three DESs from three classes, including (i) Type III, urea-choline chloride (2:1); (ii) Type V, menthol-thymol (1:1); and (iii) Type VI, menthol-ibuprofen (3:1), were prepared and characterized by ATR-FTIR spectroscopy, which supported the formation of DESs. The PCA of the FTIR data revealed that the two methods produced DESs with similar composition, as the score plots of the DESs overlapped in all cases of DES preparation. The ^1^H NMR experiment confirmed satisfactory purity of the individual compounds, i.e., urea, choline chloride, menthol, thymol, and ibuprofen. The ^1^H NMR data for the DESs prepared in both methods suggest that mechanochemical preparation of DES leads to products of higher purity. DSC and TGA-IR performed the thermal analysis of the DESs and showed exothermic transitions in the temperature range studied for all of the DESs examined in this study, but no endothermic phase transition was observed. In each case of preparation, both thermally and mechanochemically prepared DES exhibited similar patterns of freezing and crystallization of the components, apart from Thymol-Menthol DESs that have higher thermal stability than those of their individual components. The dynamic TGA curves of all the DESs tested in this study showed that the thermal stability of the DESs was between those of their individual components. For all the DES made by the thermal and mechanochemical methods in each case, the dynamic TGA curves of the DESs showed closely similar trends, which projects the mechanochemical method as an eco-friendly approach suitable for DES preparation. The combination of DSC data and TGA results suggests that the lower decomposition temperature of mechanochemically prepared DESs is likely due to a less organized molecular structure with weaker hydrogen bonding networks compared to that of thermally prepared DESs. The results from pH, Density, and Conductivity of DES's physical properties of DES are well preserved in the mechanochemical method compared to the thermal method. The results from this study portray the rotary tumbler ball milling mechanochemical method as an alternative means of preparing DES and the possibility to further tune this method for the large-scale preparation of DES, which is particularly appealing for therapeutic and pharmaceutical industries. Our future is to explore the preparation of more kinds of DES using mechanochemical methods and to better optimize them to enhance their potential for efficient DES preparation. Moreover, large-scale mechanochemical preparation of DES can be explored with a Planetary ball mill, and a semicircular and omnidirectional planetary ball machine, although such laboratory-based instruments are expensive and require external funding support.

## Supplementary Material



## Notes

Thermal DES preparation method details, ^1^H-NMR spectra and DSC data for menthol-thymol DES, stepwise figures for DES prepared in both the thermal and mechanochemical method, and FTIR spectra for Men-Ibu in varying ball bearing number (PDF)
