# Supplementary material for: A Comparative Study on Mechanochemically and Thermally Prepared Deep Eutectic Solvents
Source: ACS Omega. 2025 May 28;10(22):23335–47. doi: 10.1021/acsomega.5c01788 (PMC12163812; doi:10.1021/acsomega.5c01788)
Supplement: Supplementary file 1 [file ao5c01788_si_001.pdf]

Supporting Information

A Comparative Study on Mechanochemically and Thermally Prepared Deep Eutectic Solvents

Oluseyi Olawuyi[1], Md Rakibul Hasan[2], Tomasz Kruczyński[1], Abdul Hannan[1], Mohammad A. Halim[1]

[1]Department of Chemistry and Biochemistry, Kennesaw State University, Kennesaw, GA, 30144 USA

[2]Division of Environmental and Green Chemistry, The Red-Green Research Center, BICCB, 16, Tejkunipara, Tejgaon, Dhaka 1215, Bangladesh

*Corresponding Author: Mohammad A. Halim, Email: mhalim1@kennesaw.edu; Phone: 1-470-578-6759

Preparation of the DESs by Thermal Method

Three DESs from three classes of DESs including (i) Type III - Urea-Choline Chloride (2:1 ratio); (ii) Menthol-Thymol (1:1 ratio); and (iii) Type VI - Menthol-Ibuprofen (3:1 ratio), were synthesized using the thermal method. They were prepared at the stated molar ratio mixing the two components in each DES. Then the mixtures were heated between 40-60 °C in covered glassware for 1-2 hours under constant stirring (≤ 600 rpm) at atmospheric pressure until a homogeneous liquid was formed in each case. After the formulation, the DESs were preserved in a desiccator for further use.

^{1}H Nuclear Magnetic Resonance (NMR)

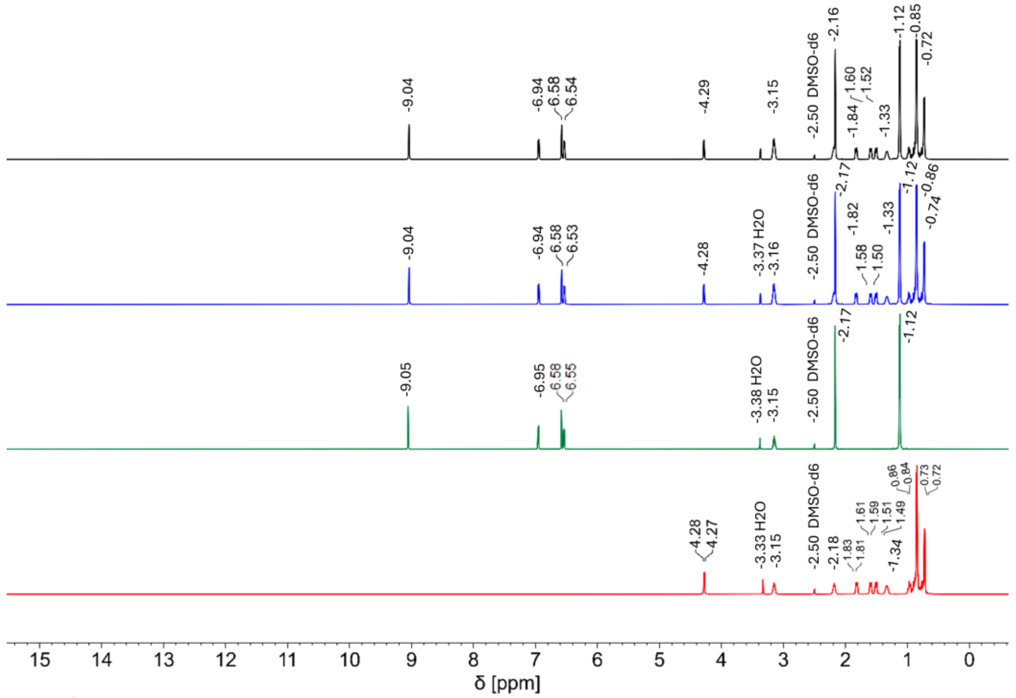

Figure S1 - ^{1}H NMR spectrum of: red – menthol; green - thymol; blue – mechano DES (1:2); black - thermal DES (1:2), DMSO-d6, 298.1 K, (^{1}H) 600.2 MHz);

Differential Scanning Calorimetry (DSC)

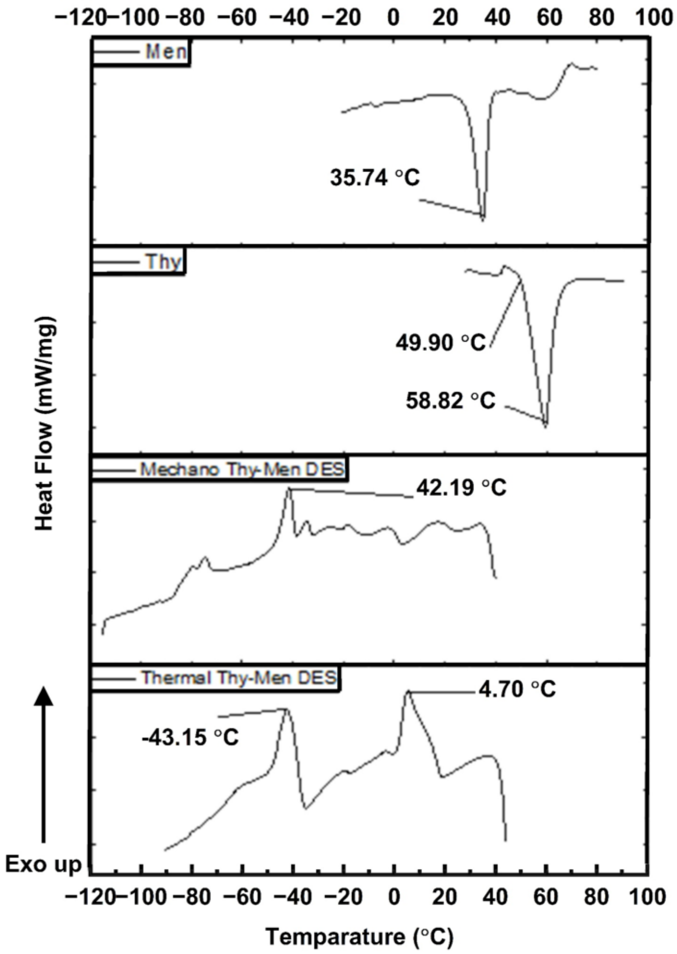

Figure S2: DSC Thermogram for Menthol, Thymol, Thermal-DES, and Mechano-DES of Men-Thy;

Synthesis of Type III Choline Chloride - Urea (1:2)

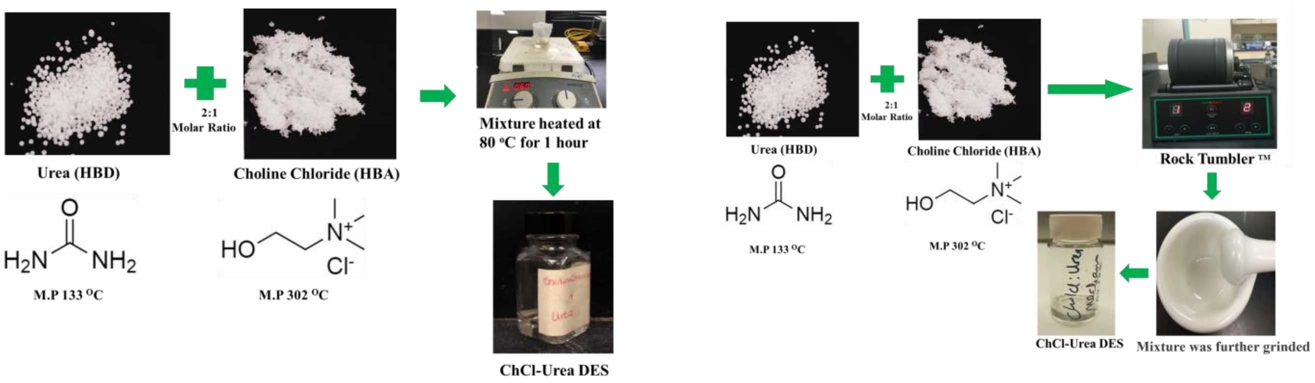

Figure S3: Synthesis of Urea-ChCl (2:1) DES by thermal (left) and mechanochemical method (right).

Synthesis of Type V Thymol - Menthol (5:5) DES

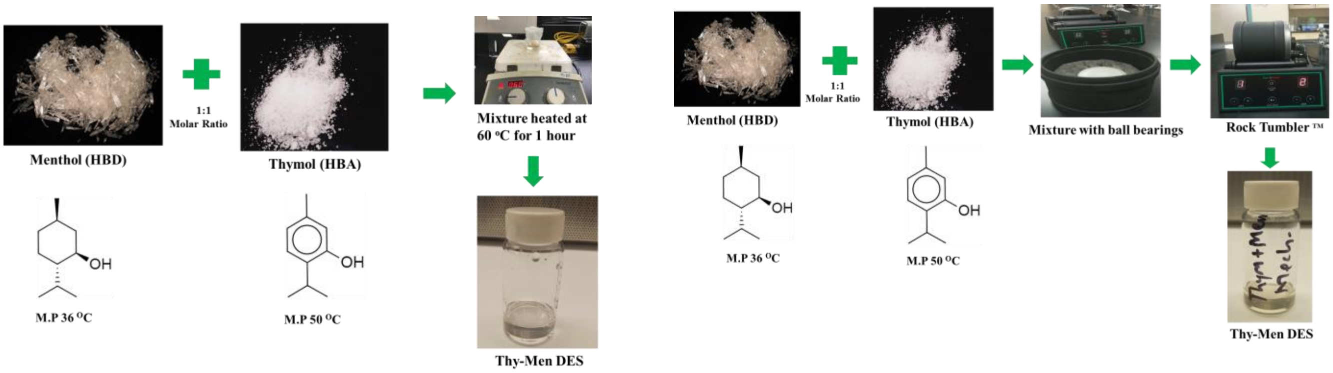

Figure S4: Synthesis of Thy-Men (5:5) DES by thermal (left) and mechanochemical method (right).

Synthesis of Type VI Ibuprofen-Menthol DES (1:3)

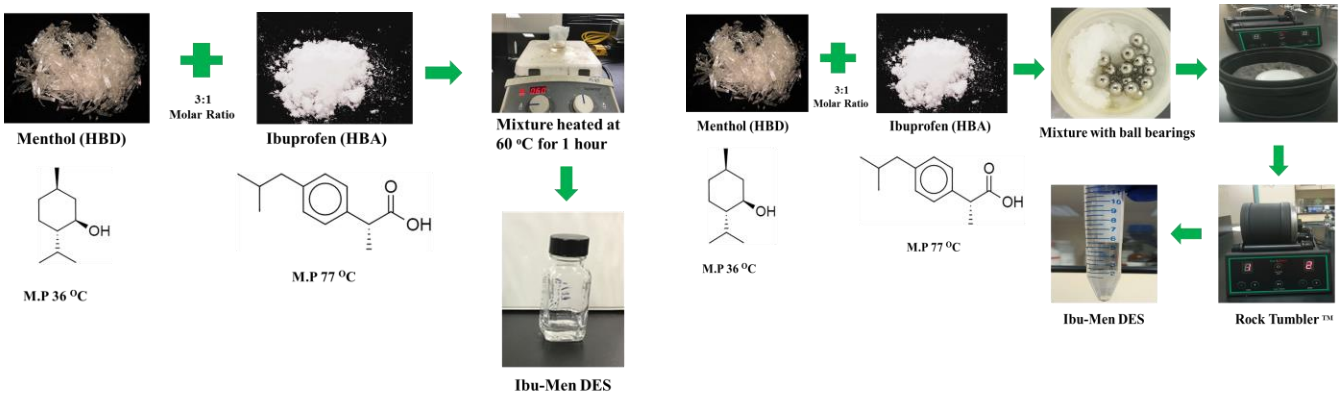

Figure S5: Synthesis of Ibu-Men (1:3) DES by thermal (left), and mechanochemical (right) methods.

FTIR spectra of menthol-Ibuprofen DES synthesized using various number of ball bearings in mechanochemical method.

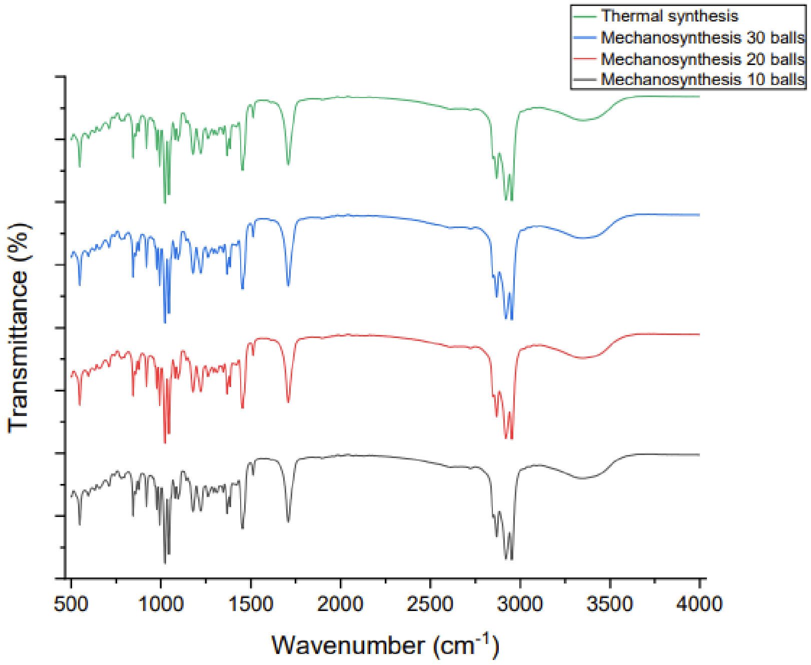

Figure S6: FTIR spectra of Ibu-Men (1:3) DESs synthesized using various number of ball bearing in mechanochemical methods.

DES	Components	Approximate amount (g)
Urea : Choline Chloride - (2:1)	Urea	1.2012
	Choline Chloride	1.3962
Menthol - Thymol (1:1)	Menthol	3.1214
	Thymol	3.0458
Menthol - Ibuprofen (3:1)	Menthol	4.6881
	Ibuprofen	2.0629

Table S1: Amount of sample required for preparation of DESs.
